# Predictors of Dropout From Residential Treatment for Posttraumatic Stress Disorder Among Military Veterans

**DOI:** 10.3389/fpsyg.2019.00362

**Published:** 2019-02-26

**Authors:** Noelle B. Smith, Lauren M. Sippel, David C. Rozek, Rani A. Hoff, Ilan Harpaz-Rotem

**Affiliations:** ^1^VA Northeast Program Evaluation Center, West Haven, CT, United States; ^2^Department of Psychiatry, School of Medicine, Yale University, New Haven, CT, United States; ^3^National Center for PTSD, White River Junction, VT, United States; ^4^Department of Psychiatry, Geisel School of Medicine at Dartmouth, Hanover, NH, United States; ^5^Department of Psychiatry, University of Utah, Salt Lake City, UT, United States; ^6^National Center for Veterans Studies at the University of Utah, Salt Lake City, UT, United States; ^7^National Center for PTSD, VA Connecticut Healthcare System, West Haven, CT, United States

**Keywords:** treatment failure, PTSD, veterans, residential, psychotherapy, drop-out, program completion

## Abstract

**Background:** Successful psychotherapy for posttraumatic stress disorder (PTSD) necessitates initial and sustained engagement. However, treatment dropout is common, with rates of 50–70% depending on the setting, type of treatment and how dropout is calculated. Dropout from residential treatment is less understood and could be impacted by participation of more symptomatic patient populations and reduced day-to-day barriers to engagement. Gaining insight into predictors of treatment dropout is critical given that individuals with greater symptoms are the most in need of successful treatments but also at higher risk of unsuccessful psychotherapy episodes.

**Aim:** The aim of the current study was to examine predictors of treatment dropout among veterans receiving residential treatment for PTSD.

**Methods:** The study included 3,965 veterans who initiated residential PTSD treatment within a Department of Veterans Affairs program during Fiscal Year 2015 and completed self-report measures of demographics and psychiatric symptoms at admission.

**Results:** In our sample (*N* = 3,965, 86.5% male, mean age = 45.5), 27.5% did not complete the residential program (*n* = 1,091). Controlling for age, marital status, combat/non-combat trauma, and facility, generalized estimating equation modeling analysis indicated greater PTSD symptoms and physical functioning at admission were associated with reduced likelihood of completing the residential program. There were significant differences in trauma-focused psychotherapy received by individuals who dropped out of residential treatment and those who did not. Among veterans who dropped out, 43.6% did not get any trauma-focused psychotherapy; 22.3% got some, but less than 8 sessions; and 34.1% got at least 8 sessions; compared to 37.3%, 4.8%, and 57.9%, respectively, among program completers.

**Conclusion:** Dropout rates from residential PTSD programs indicate that at least one in four veterans do not complete residential treatment, with more symptomatic individuals and those who do not receive trauma-focused therapy being less likely to complete.

## Introduction

Rates of posttraumatic stress disorder (PTSD) are considerably high among United States military veterans (e.g., Hoge et al., 2004; Kok et al., 2012; Fischer, 2015). In response to these high rates of PTSD, there has been attention paid to the delivery of evidence-based treatment to veterans with PTSD, particularly at the Department of Veterans Affairs (VA). There are many evidence-based treatments for PTSD that are efficacious among veterans. Recent clinical practice guidelines identified trauma-focused psychotherapies (TFP) as the first-line treatment for PTSD (Veterans Affairs Department of Defense, 2017), which can be delivered in a variety of settings (e.g., outpatient, residential). To date, much of the extant literature has focused on examining treatment outcome in outpatient settings, leading to ongoing questions regarding treatment success or failure in residential treatment settings. It is imperative to understand predictors of unsuccessful treatment for this population so that the field may more effectively intervene to maximally facilitate successful outcomes.

A given course of treatment can be considered unsuccessful if (1) a patient does not engage in the treatment, such as not attending a first session after being assessed, referred, and consented to treatment; (2) a patient initially engages in treatment but prematurely discontinues before completing a full course or dose of treatment; or (3) a patient engages in treatment but it is not effective for symptom severity and functional outcomes even when delivered at an adequate dose and with good fidelity (Sippel et al., 2018). This current examination will focus on (2): premature termination of PTSD treatment delivered in residential treatment settings through the examination of rates of residential program completion and whether completion is predicted by the receipt of trauma-focused (i.e., evidence-based) psychotherapy.

Treatment dropout has gained attention in both randomized clinical trials and real-world clinical care, with some individuals beginning but not completing a full course of treatment and therefore not having the opportunity to maximally benefit. Meta-analytic findings indicate that, across psychological disorders, roughly 20% of patients prematurely terminate psychotherapy (Swift and Greenberg, 2012), with higher odds of dropout from pharmacotherapy than psychotherapy (Swift et al., 2017).

Extant research on treatment dropout in PTSD is based on randomized clinical trials of specific psychotherapies typically delivered in outpatient settings or effectiveness data from outpatient clinics (Goetter et al., 2015). Rates of dropout from psychotherapy for PTSD vary widely, with meta-analytic findings identifying an average dropout rate of 18% in randomized clinical trials (Imel et al., 2013). Rates tend to be higher in naturalistic clinic-based studies, with a pooled average of 42.0% in these settings (Goetter et al., 2015). However, dropout rates are not higher in trauma-focused vs. non-trauma-focused therapies (Imel et al., 2013). Patient-related predictors of dropout have included younger age (e.g., Erbes et al., 2009; Garcia et al., 2011; Kehle-Forbes et al., 2016; Goodson et al., 2017; Niles et al., 2018) and higher PTSD symptom severity (Garcia et al., 2011; Grubbs et al., 2015), though some studies have not found that PTSD severity is associated with dropout (Kehle-Forbes et al., 2016; Niles et al., 2018). When dropout occurs, it tends to be early in treatment, around sessions two to four (e.g., Garcia et al., 2011; Davis et al., 2013; Mott et al., 2014; Kehle-Forbes et al., 2016).

To our knowledge there are no existing published studies examining dropout from residential treatment programs for PTSD. However, there have been examinations of associations between length of stay in residential treatment and clinical outcomes among veterans. For example, homeless women veterans who received greater than 30 days of residential treatment exhibited more improvement in mental health symptoms and functional outcomes at one-year follow-up than veterans who received fewer than 30 days of treatment (Harpaz-Rotem and Rosenheck, 2011). Longer length of stay has also predicted better outcomes for common comorbidities such as alcohol misuse (Harpaz-Rotem and Rosenheck, 2011; Coker et al., 2016). A recent study revealed that longer length of stay in residential treatment programs at five VA facilities was associated with more severe PTSD symptoms at baseline and less severe PTSD symptoms at discharge (Banducci et al., 2017). Among individuals who did not complete an inpatient PTSD program, a shorter stay was related to less symptom improvement (Szafranski et al., 2014). Taken together, it appears that longer courses of residential treatment may be associated with better outcomes among veterans, though there is limited evidence focused specifically on PTSD and significant heterogeneity in the clinical programming and duration of residential programs.

Examination of dropout from a residential setting is critical, as it is considered a higher level of care and thus oftentimes attracts a more symptomatic population. As indicated previously, more symptomatic individuals may be more likely to drop out of treatment delivered in outpatient settings (Garcia et al., 2011; Grubbs et al., 2015), making this a particularly vulnerable population. However, residential treatment is associated with fewer logistical barriers to completion such as reduced day-to-day stressors at home/work, transportation, and housing. Thus, it is critical to determine potential risk factors for premature termination from both residential treatment and trauma-focused psychotherapy in order to address these factors and enhance treatment completion and outcome.

This is the first study to our knowledge to examine rates and predictors of premature termination (i.e., dropout) from VA residential PTSD programs. The overarching goal of this study was to better understand this form of treatment failure so that the field may better address these predictors to bolster treatment completion. Our first aim was to characterize rates of dropout in this population and to determine differences between individuals who prematurely terminate and those who complete treatment. We hypothesized that the rate of dropout would be lower than rates published with samples from outpatient settings, potentially due to reduced logistical barriers. Our second aim was to determine bivariate correlates as well as predictors (e.g., demographics, clinical characteristics) of residential treatment dropout in multivariate analyses after accounting for data nested within sites. We hypothesized that more severe symptoms would be associated with dropout, consistent with prior research in outpatient settings. Our third aim was to examine receipt of TFP among individuals who dropped out of residential treatment in order to enhance our understanding of whether engagement in first-line treatment is associated with reduced risk of dropout.

## Materials and Methods

### Participants and Procedure

The study included 3,965 veterans who initiated residential PTSD treatment within a Department of Veterans Affairs residential treatment program during Fiscal Year 2015 (FY15) and completed self-report measures of demographics and psychiatric symptoms at admission. Program clinicians and staff completed measures indicating a veteran’s completion of the residential program, as well as information regarding the dose of trauma-focused psychotherapy each veteran received, at discharge. This study was approved by the VA Connecticut Healthcare System Institutional Review Board.

### Measures

#### Demographic Information

Demographic information was collected, including: age, sex, race, ethnicity, and marital status. Experience of combat trauma was also assessed, as well as the site at which the veteran participated in residential treatment.

#### Treatment Completion

Clinicians indicated whether each veteran completed residential treatment or if they dropped out. Clinician-rated premature termination has been employed in previous studies examining discontinuation of treatment (e.g., Garcia et al., 2011). Program length and services offered vary by site; thus, treatment completion was determined by clinical staff at each site. This variable was dichotomized (dropped out: 0; completed: 1).

#### Receipt of Trauma-Focused Psychotherapy

Clinicians also indicated the extent to which individuals received trauma-focused treatment defined by the protocol-based number of sessions. Individuals who received eight or more sessions were considered to have completed TFP, whereas those who completed less than 8 were characterized as not receiving a TFP. Eight sessions in 14 weeks is a rough metric intended to capture participation in an evidence-based treatment such as TFPs in previous studies (VA Office of Inspector General, 2012) and many participants meet end-state criteria by session 8 (e.g., Galovski et al., 2012); thus, this number of sessions was selected as indicating receipt of TFP. For our third aim, this variable was dichotomized (less than 8 sessions: 0; 8 or more sessions: 1) to better understand dropout from TFP.

#### PTSD Symptoms

At admission, veterans completed the PTSD Checklist-5 (PCL-5; Weathers et al., 2013), a 20-item self-report measure that assesses severity of PTSD symptoms according to the Diagnostic and Statistical Manual of Mental Disorders-Version 5 (DSM-5; American Psychiatric Association, 2013) diagnostic criteria. Higher scores reflect greater PTSD scores. It had good reliability in the current sample (α = 0.91).

#### Substance Use

At admission, veterans completed the Brief Addiction Monitor (BAM; Cacciola et al., 2013), a 17-item, multi-dimensional questionnaire designed to assess frequency of substance (alcohol and drug) use. In this study, we used the three items that sum the total amount of substances used in the past 30 days (alcohol, illegal drugs, and prescribed medication). A score of 0 reflects 0 days; 1 = 1–3 days; 2 = 4–8 days; 3 = 9–15 days; and 4 = 16–30 days, with higher scores reflecting more frequent substance use.

#### Physical and Mental Health

At admission, veterans completed items of the Short Form Health Survey (SF-12; Ware et al., 1996); these components were used to assess various domains of mental and physical health. Physical functioning and role-physical were components of physical health that were included, while role-emotional represented mental health, as these were the components that appear to best predict physical and mental health. Scores on the SF-12 were transformed to z-scores using means and standard deviations from the general population to account for population-based norms (Ware et al., 1996). Higher scores reflected better health.

### Data Analytic Strategy

Prior to all analyses, descriptive statistics were run to identify means and standard deviations for the study variables. To address aim 1, we conducted descriptive statistics and tests of difference (*t*-tests and chi-squared tests) for completers and non-completers (i.e., those who dropped out). To address aims 2 and 3, we ran bivariate correlations to identify significant associations between predictor variables (e.g., demographics and clinical characteristics) and outcome variables (aim 2: treatment completion; aim 3: receipt of TFP) for all veterans. We then used multivariate Generalized Estimating Equation (GEE) modeling to examine the relation between significant demographic and clinical characteristics and program completion vs. dropout in the first analysis and receipt of TFP in the second. GEE was used to adjust for correlated observations (Liang and Zeger, 1993), such as data nested within residential sites. The PROC GENMOD procedure of SAS was used for these analyses and probabilities were modeled for those who dropped out and those who did not receive TFP, respectively.

## Results

Our sample (*N* = 3,965) was predominantly male (86.5%), with a mean age of 45.54 (standard deviation = 13.38). Table 1 includes demographic and clinical characteristics of study participants.

**Table 1:** Demographic and clinical characteristics of the total sample and each group (Program completers and non-completers).

	Total	Completers	Non-completers	Test of difference
	*N* = 3,965	*n* = 2,874	*n* = 1,091	
Age, *M* (*SD*)	45.54 (13.38)	46.23 (13.43)	43.65 (13.07)	*t =* -5.51*, p* < 0.001
Ethnicity: white, *n* (%)	2,340 (59)	1,698 (59.1)	652 (58.8)	χ^2^(1) = 0.02, *p* = 0.89
Military trauma, *n* (%)	3,746 (94.5)	2,710 (94.3)	1,036 (95)	χ^2^(1) = 0.67, *p* = 0.41
Married, domestic partner *n* (%)	1,580 (39.8)	1,189 (41.7)	391 (36.1)	χ^2^(1) = 10.53, *p* = 0.05
Sex: male, *n* (%)	3,430 (86.5)	2,488 (89)	942 (89.6)	χ^2^(1) = 0.27, *p* = 0.61
TFP (8+ sessions), *n* (%)	2,019 (50.1)	1,663 (57.9)	356 (34.1)	χ^2^(1) = 173.15, *p* < 0.001
Substance use^1^, *M* (*SD*)	2.46 (3.04)	3.01 (0.56)	3.11 (0.09)	*t =* 1.46, *p =* 0.14
PTSD symptoms (PCL-5), *M* (*SD*)	58.93 (11.97)	58.57 (12.23)	59.90 (11.22)	*t =* 3.13, *p =* 0.002
Physical functioning (SF-12), *M* (*SD*)	-0.86 (1.22)	-0.90 (1.23)	-0.76 (1.21)	*t =* 3.26, *p =* 0.001
Role-physical (SF-12), *M* (*SD*)	-1.30 (1.13)	-1.32 (1.13)	-1.26 (1.13)	*t =* 1.40, *p =* 0.16
Role-emotional (SF-12), *M* (*SD*)	-2.40 (1.07)	-2.39 (1.07)	-2.44 (1.06)	*t =* -1.36, *p =* 0.17


*^*1*^Includes items assessing substance use on the BAM Brief Addiction Monitor (BAM; Cacciola et al., 2013). TFP, Trauma-focused psychotherapy; PCL-5, Posttraumatic stress disorder checklist-5 (PCL-5; Weathers et al., 2013); SF-12, Short form health survey (SF-12; Ware et al., 1996)*.

Our first aim was to characterize dropout among veterans in residential PTSD treatment. In our sample, 27.5% did not complete the residential program (*n* = 1091). Table 1 displays group differences between residential program completers and those who dropped out. Individuals who dropped out were younger, had more severe PTSD symptoms, and reported better physical functioning. There were significant differences in receipt of TFP by individuals who dropped out of residential treatment and those who did not, χ^2^(2) = 338.17, *p* < 0.001. Among veterans who dropped out, 65.9% got less than eight sessions; and 34.1% got at least eight sessions; compared to 42.1% and 57.9%, respectively, among program completers.

Our second aim was to identify correlates and predictors of program completion. Bivariate correlations indicated significant relations between program completion and age (*r* = 0.09, *p* < 0.001), marital status (*r* = 0.05, *p* = 0.001), PTSD symptoms (*r* = -0.05, *p* = 0.002), and physical functioning (*r* = -0.05, *p* = 0.001). Including these significant variables in the model and after controlling for data nested within sites, GEE analyses indicated that age, PTSD symptoms, and physical functioning were significantly related to program completion, such that younger age, greater PTSD symptoms, and better physical functioning were associated with reduced likelihood of completing the program. See Table 2 for a summary of the GEE analysis.

**Table 2:** Results from generalized estimating equation analysis predicting residential treatment non-completion.

*Parameter*	*Estimate*	Standard error	95% confidence limits		*Z*	Pr Z > |Z|
Intercept	-0.41	0.15	-0.70	-0.11	-2.72	0.007
Age	-0.01	0.00	-0.02	-0.01	-4.46	<0.001
Married	0.14	0.09	-0.03	0.31	1.59	0.11
Non-combat trauma	-0.16	0.17	-0.50	0.28	-0.92	0.36
PTSD symptoms (PCL-5)	0.09	0.03	0.03	0.15	2.86	0.004
Physical functioning (SF-12)	0.06	0.03	0.01	0.11	2.30	0.022


*This analysis was run modeling the probabilities of dropout/non-completers (0 dropout, 1 complete). PCL, standardized scores from posttraumatic stress disorder checklist-5 (PCL-5; Weathers et al., 2013); SF-12, short form health survey (SF-12; Ware et al., 1996)*.

Our third aim was to examine receipt of TFP among those who dropped out. Among individuals who did not complete residential treatment, bivariate correlations indicated significant relations between TFP completion and ethnicity (*r* = -0.066, *p* = 0.033) and alcohol use (*r* = -0.082, *p* = 0.008). Results of GEE indicated that neither variable (ethnicity: *B* = -0.20, 95% Confidence interval = -0.03, 0.05, *p* = 0.35) or alcohol use: (*B* = 0.01, Confidence interval = -0.62,.22, *p* = 0.64) was significantly associated with outcome when in the multivariate analysis when accounting for nesting with sites.

## Discussion

This was the first study to examine predictors of treatment dropout among a national sample of veterans who engaged in residential PTSD treatment. We found that over one in four veterans prematurely terminated residential treatment. Although this number is lower than in outpatient settings (e.g., Garcia et al., 2011; Kehle-Forbes et al., 2016; Doran and DeViva, 2018), it is alarming that a significant minority of veterans are prematurely terminating treatment despite reduced logistical barriers such as need to travel and work obligations. We discovered that younger age, more severe PTSD symptoms, and better physical functioning were related to premature termination of residential PTSD treatment.

Our results are consistent with previous studies in outpatient settings indicating that younger age is associated with greater likelihood of dropout (e.g., Garcia et al., 2011; Kehle-Forbes et al., 2016; Goodson et al., 2017; Niles et al., 2018). Although previous literature is mixed as to whether PTSD symptoms are associated with dropout (e.g., Garcia et al., 2011; Grubbs et al., 2015; Kehle-Forbes et al., 2016; Niles et al., 2018), we found support for the premise that individuals with more symptoms at baseline were less likely to complete residential treatment. Identifying younger and more symptomatic veterans at admission could be important in reducing dropout. Creating interventions to enhance engagement for these individuals may be key to promoting successful treatment.

The findings from this study have important clinical implications as they indicate that those who are more in need of treatment (e.g., those with higher symptoms) are more likely to drop out from residential programs. Although initial impairment appears to be a general risk factor for premature termination in our study as well as in outpatient samples with various diagnoses (e.g., Zimmermann et al., 2017), there are potentially ways to target this group. Additionally, adherence to TFP protocols, veterans’ agency in treatment choice, and attitudes regarding treatment effectiveness (Zimmermann et al., 2017; Doran and DeViva, 2018; Zoellner et al., 2018) could be ways to address internal barriers to completion. Including motivational enhancement techniques in this particularly at-risk group is a potential avenue to address premature termination (Murphy et al., 2009). An additional option is to target provider characteristics, such as enhancing training in TFPs, which has been shown to be related to reduced treatment dropout (e.g., Goodson et al., 2017). A third option is to increase utilization of virtual reality as it appears to have lower dropout rates (Benbow and Anderson, 2018), and to be efficacious in the treatment of PTSD (Gonçalves et al., 2012). Finally, increasing the availability and delivery of TFPs and in ways that have been shown to increase the completion rate of treatment such as condensed daily sessions of treatment (e.g., Bryan et al., 2018; Foa et al., 2018) or perhaps by combining a condensed protocol with virtual reality (e.g., Beidel et al., 2017). This would allow for patients to complete TFP as part of their daily residential routine with the same outcomes as weekly treatment. These options offer promising ways to enhance the care that veterans receive in residential treatments in order to increase completion of TFPs and reduce the impact of PTSD on long-term outcomes.

It was surprising that better physical functioning was related to greater treatment dropout. It could be that those with reduced functioning tended to rely more on the services provided in a residential setting thus attenuating tendencies to prematurely terminate. Alternatively, those with greater physical functioning could more readily apply some of the techniques that might be helpful in reducing symptoms such as physical activity (Rosenbaum et al., 2015), behavioral activation (e.g., Jakupcak et al., 2010), and *in vivo* exposure techniques (e.g., Gros et al., 2012).

We also found that the individuals who dropped out of the program were less likely to receive at least eight sessions of TFP, which is expected since dropout typically occurs in earlier sessions (Garcia et al., 2011; Davis et al., 2013; Mott et al., 2014; Kehle-Forbes et al., 2016). Among non-completers, there were significant correlates of receipt of TFP, but none of these variables predicted receipt of TFP in multivariate analyses. However, given bivariate relations, it could be important to consider pre-treatment substance use as a potential additional risk factor for not receiving at least eight sessions of TFP, particularly given that there appears to be a bidirectional relationship between PTSD symptoms and substance use (Back et al., 2014) and that substance use is related to a shorter length of stay in inpatient settings (Szafranski et al., 2014).

It is important to highlight that it is unlikely that veterans dropped out of treatment due to reduced symptoms (e.g., Szafranski et al., 2017). Only 34.1% of those who dropped out received eight or more sessions of a TFP, which is thought to be the point at which end state criteria are met; in comparison, more than half of the completers (57.9%) received eight or more sessions. The majority of individuals who dropped out did not receive a substantial course of TFP; this is consistent with data indicating that many veterans with PTSD are not receiving clinically adequate care (i.e., at least eight appointments in 14 weeks; Smith et al., 2017). However, our results indicate that when veterans complete residential programming, the majority of veterans received eight or more sessions of TFP. Therefore, addressing premature termination could help to enhance the proportion of individuals receiving gold standard treatment.

The quality of treatment program or therapies delivered was not assessed in the current study and there appears to be varying degrees of adoption of TFP among various residential programs (Cook et al., 2013). It was striking that almost half (43.6%) of those who dropped out did not receive any TFP. Even among treatment completers, more than 1/3 did not receive any sessions of the gold standard treatments. Thus, there appears to be room for enhanced adoption and delivery of TFPs among residential programs.

This study has a number of limitations that require mentioning. Clinician-reported completion of program and TFP limited understanding of veterans’ perspective on treatment completion. Previous receipt of treatment for PTSD in outpatient or residential settings, length of time since trauma or PTSD diagnosis, and information related to deployment or childhood trauma were not available in the current study, but are important, as these factors could impact treatment dropout, compliance, and participation in TFP. Dichotomized outcomes did not account for dose of clinical programming or dose of TFPs. Additionally, VA residential treatment programs have significant heterogeneity in program length and composition; therefore it is difficult to compare across treatment programs. Although we controlled for data nested within sites, we did not control for other differences across the sites. Moreover, symptoms were not assessed at the time of program termination and veteran self-reported reasons for not completing the program were not assessed, thus limiting the understanding of treatment dropout.

This study provides a preliminary investigation into components of dropout as an important form of treatment failure in the residential PTSD treatment programs. More symptomatic veterans appear to be at increased risk of premature termination, consistent what has been observed in outpatient populations. Future studies should qualitatively investigate veterans’ reasons for treatment dropout, examine the role of medication usage and compliance, determine symptom changes across the course of treatment and how they relate to program completion, and examine moderators of treatment completion (e.g., Keefe et al., 2018) and receipt of TFPs in order to enhance treatment completion and outcome.

## Author Contributions

NS was involved in idea generation, data analysis, manuscript preparation, and submission preparation. LS and DR reviewed the literature and contributed to manuscript preparation, editing, revising, and table construction. RH contributed to data preparation and various components of manuscript preparation. IH-R was involved in idea generation, prepared and analyzed the data, and contributed to manuscript preparation.

## Disclaimer

The content is solely the responsibility of the authors and does not necessarily represent the official views of the United States Department of Veterans Affairs.

## Conflict of Interest Statement

The authors declare that the research was conducted in the absence of any commercial or financial relationships that could be construed as a potential conflict of interest.
